# A review of sperm cryopreservation in the domestic dog and cat—part II, freezing epididymal spermatozoa, why is it different from ejaculated spermatozoa?

**DOI:** 10.1186/s13028-025-00846-1

**Published:** 2025-12-11

**Authors:** Eva Axnér

**Affiliations:** Department of Clinical Sciences, Swedish University of Agricultural Sciences, P.O. Box 7054, Uppsala, 750 07 Sweden

**Keywords:** Artificial insemination, Canine, Comparative reproduction, Cryobiology, Cryopreservation, Feline, Reproduction, Sperm

## Abstract

If a male dies suddenly or requires castration, it may still be possible to produce offspring through artificial insemination (AI) by cryopreserving spermatozoa retrieved from the epididymis. Spermatozoa differ in maturation status along the epididymal duct, and only motile spermatozoa that have acquired fertilizing capacity are suitable for AI. Such spermatozoa can be collected from the terminal epididymal segment, located in the cauda epididymidis in the dog, and in both the corpus and cauda in the cat. Unlike ejaculated spermatozoa, epididymal spermatozoa have not been exposed to seminal plasma and therefore display distinct functional and structural characteristics. The method of sperm collection may also affect the sperm quality. While epididymal mincing results in the highest sperm numbers, it is associated with contamination of blood and epididymal tissue. Although numerous studies have reported successful cryopreservation of epididymal spermatozoa in dogs and cats, reports of live offspring following AI with frozen–thawed epididymal spermatozoa remain scarce. This review summarizes the physiological, anatomical, and functional distinctions between epididymal and ejaculated spermatozoa, emphasizing their implications for cryopreservation strategies and fertility outcomes.

## Background

Epididymal spermatozoa can be collected following orchidectomy, or even after the death of a male. Cryopreservation of epididymal spermatozoa therefore presents a valuable option in conservation biology, particularly when a male of a threatened species dies unexpectedly. The domestic dog and cat are often used as model species for wild canids and felids. Epididymal sperm collection is also relevant in clinical practice, for example when a male requires orchidectomy, or dies suddenly in an accident [1]. However, males that die or are euthanized after prolonged illness often exhibit poor semen quality (author’s own experience). Thus, epididymal sperm cryopreservation is most successful in males that were previously healthy, such as those euthanized or deceased due to acute trauma. Unlike ejaculated spermatozoa, epididymal spermatozoa have not been exposed to seminal plasma. The mixing of spermatozoa with seminal fluids at ejaculation induces maturational changes in the plasma membrane that may alter both cryotolerance and fertilizing capacity. Because orchidectomy is performed routinely in dogs and cats, epididymal spermatozoa are readily accessible for research. Therefore, there are several reports on the collection and preservation of epididymal spermatozoa from these species, while reports of live offspring derived from frozen–thawed epididymal spermatozoa remain rare. As cryopreservation methods for epididymal sperm generally follow those established for ejaculated samples, detailed descriptions of freezing protocols are not included here, as they have been reviewed elsewhere [2].

## Search strategy

This narrative overview is based on a search in PubMed (https://pubmed.ncbi.nlm.nih.gov/) and Web of Science (https://www.webofscience.com/wos/alldb/basic-search) using the terms “artificial insemination, canine, cold shock, cryocapacitation, cryopreservation, equilibration, extenders, epididymal, feline, freezing, semen, sperm, spermatozoa.” In addition, the author’s own archive on ”Endnote TM^20^ (Clarivate analytics)” was used as a source.

## Review

### The epididymal duct

**Fig. 1:**
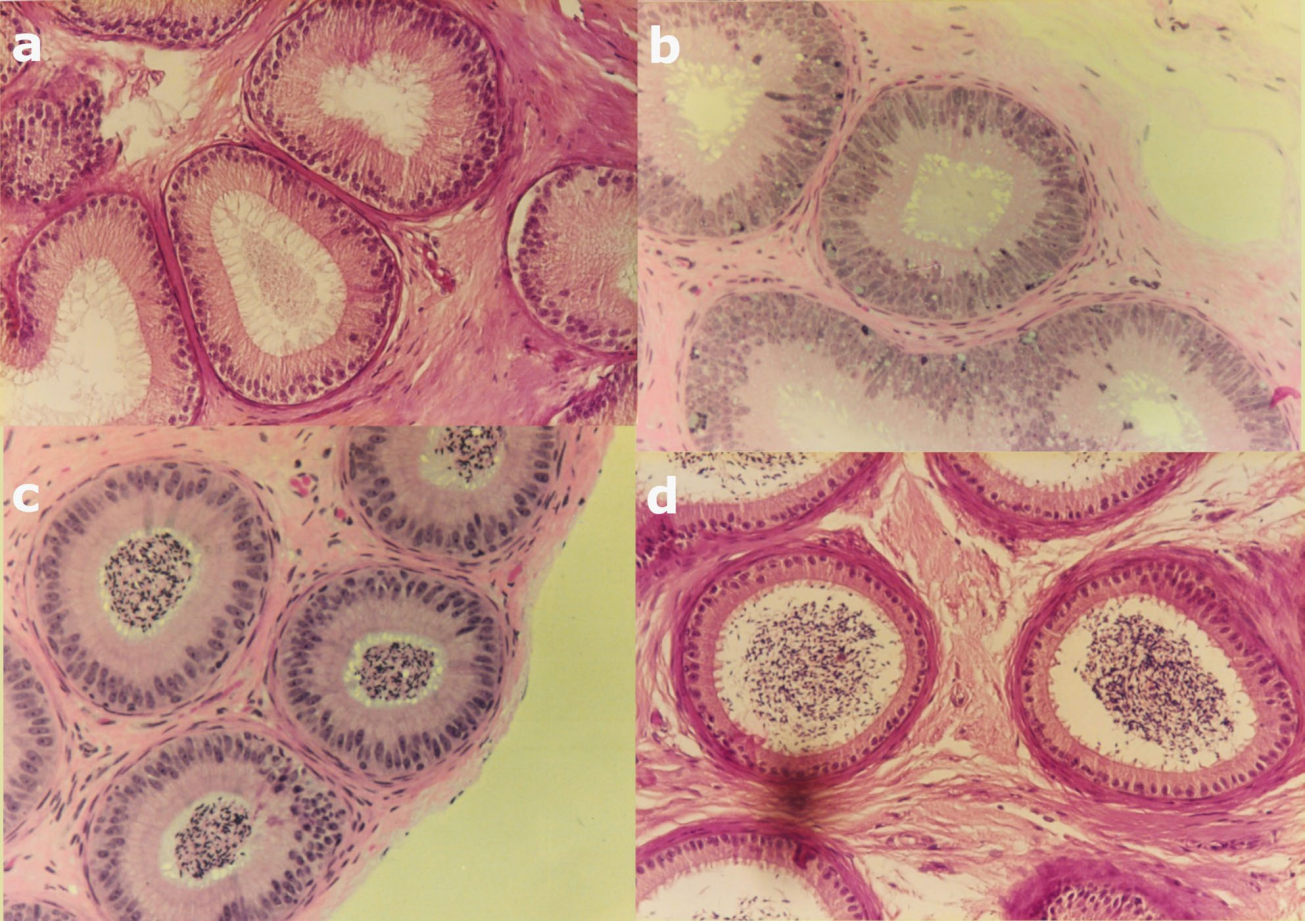
Histology of the feline epididymis, x 200, stained with haematoxylin and eosin. **a** Histology of region 1, corresponding to the initial segment, **b** Histology of region 2, the first part of the middle segment. Vesicles are abundant in the cytoplasm, **c** Histology of region 3, the middle part of the middle segment in the feline epididymis. The concentration of spermatozoa in the lumen is higher than in the previous region. This region has the smallest diameter of the duct in the feline epididymis. **d** Region 6 situated in the cauda epididymidis. The lumen has the highest diameter, and the epithelium is the lowest in the feline epididymal duct. Short stereocilia and a lumen filled with spermatozoa are characteristic typical of the terminal segment

The epididymis is a single long convoluted duct, traditionally divided into caput, corpus and cauda based on gross anatomy. A functional division into initial, middle and terminal segments has also been described. The initial segment is primarily involved in fluid absorption [3], the middle segment in sperm maturation, and the terminal segment in sperm storage prior to ejaculation [4]. However, the functional segmentation does not necessarily correspond to the macroscopic divisions in all species. For purposes of sperm conservation, functional segmentation may be more relevant than anatomical division. Histologically, the initial segment is characterised by tall principal cells with tall sterocilia (Fig. 1a), the middle segment by supranuclear vacuolisation (Figs. 1b and c), and the terminal segment by a short epithelium, with short sterocilia and a wide lumen (Fig. 1d) [4, 5]. In the cat, the initial segment is localized in the first part of the caput, whereas the middle segment, with pronounced secretory activity, is situated in the distal caput. Fluid resorption in this region is reflected by a sharp increase in sperm concentration from region 2 to 3 of the caput (Figs. 1b and c) [6]. The feline corpus and cauda exhibit features typical of the terminal segment [5]. In the dog, the initial segment is located in the caput, the middle segment (with both absorptive and secretory functions), is located in the corpus, and the cauda corresponds ultrastructurally to the terminal segment [4, 7]. Thus, species-specific differences exist: in dogs the middle segment is located in the corpus [7–9], whereas in cats this region is histologically more consistent with the terminal segment [5].

### Epididymal sperm maturation

Spermatozoa leaving the testis and entering the caput epididymidis are immotile and lack zona pellucida binding ability [10]. They are transported by peristaltic activity to the cauda epididymis where they are stored. During epididymal transit, the spermatozoa undergo maturational changes including acquisition of fertilising capacity. Spermatozoa from the cauda are capable of motility and can fertilize oocytes after in vitro fertilization (IVF) or artificial insemination (AI) [3, 10]. A characteristic morphological marker of maturation is the migration of the cytoplasmic droplet from a proximal to a distal position, occurring in species-specific regions. In cats, droplet migration occurs in the distal middle segment, at the caput to corpus transition [11], whereas in dogs it occurs within the corpus (Table 1) [12, 13]. Spermatozoa in the cauda epididymis typically have distal cytoplasmic droplets, which are usually shed during ejaculation when motility is activated [14]. Loss of distal droplets can also be observed when epididymal spermatozoa are collected in a media that promotes motility [11, 15, 16]. In contrast to distal droplets, proximal droplets remaining on spermatozoa in the cauda are not lost at ejaculation [6, 14]. Persistence of proximal droplets in spermatozoa in the cauda epididymidis is a marker of sperm maturation failure. Another maturational change is the decline in the proportion of spermatozoa with swollen acrosomes during epididymal transit [6, 11, 12]. Water channels, known as aquaporins, mediate movement of water and cryoprotectants across the sperm plasma membrane. In dogs, aquaporins show different immunolocalisation in capus versus cauda spermatozoa [17]. Aquaporins are probably important for the sperm cell’s response to osmotic pressure in the environment [17]. Another important maturation marker, the ability for motility, is acquired in the corpus epididymidis in the dog [13], and at the transition between the caput and corpus in the cat [11, 18, 19] (Table 1). These processes are accompanied by changes in plasma membrane phospholipid composition and cholesterol content, resulting in modified membrane fluidity [20–22]. Canine spermatozoa retrieved from the cauda epididymidis have higher concentration of saturated and unsaturated fatty acids compared with spermatozoa from the corpus and caput. The concentration of caprylic fatty acid is increased in the canine cauda, possibly contributing to greater plasma membrane integrity [20]. Also, the concentrations of antioxidants change during canine epididymal maturation. Glutathione peroxidase concentration increases from the canine caput to cauda epididymides and is higher in the cauda epididymis than in ejaculates. Catalase was found in ejaculated but not in epididymal canine sperm samples, while no significant differences between epididymal regions or between ejaculated and epididymal spermatozoa were found for superoxide dismutase [23]. The possible implications of these changes remain to be elucidated.

**Table 1:** Changes in progressive motility and the proportion of immature spermatozoa in canine and feline spermatozoa during passage through the epididymal duct

Epididymal region		Caput	Corpus	Cauda	References
Progressive motility (%)	Canine	0.0 ± 0.0	8.7 ± 1.2	33.7 ± 2.1	[23]
		0.0 ± 0.0	5.5 ± 0.9	27.8 ± 2.9	[20]
	Feline	5.3 ± 2.9	32.7 ± 4.0	51.8 ± 5.0	[18]
Proximal droplets (%)	Canine	52.2 ± 3.7	6.0 ± 1.1	0.7 ± 0.2	[13]
		44.8 ± 19.5	8.9 ± 6.8	3.6 ± 3.9	[12]
	Feline^*^	77.6 ± 9.0	17.4 ± 12.2	9.6 ± 8.9	[11]

^*^Caput = regions 1–4, calculated from data presented in [11]

During maturation, spermatozoa also develop susceptibility to cold shock. This occurs in the same epididymal region where cytoplasmic droplet migration takes place [24]. In several species, ejaculated sperm are more vulnerable to cold shock than epididymal spermatozoa [25]. During cryopreservation, spermatozoa are exposed to osmotic stress. In cats, caudal spermatozoa are more susceptible to osmotic stress than spermatozoa from the caput or corpus [26], although corpus spermatozoa show similar freezability to caudal spermatozoa [27]. While ejaculated spermatozoa often displayed higher motility, membrane integrity, and acrosome integrity both before and after cooling, source (epididymal vs. ejaculated) did not affect the susceptibility to cooling [28]. Nor did ejaculated and epididymal spermatozoa collected from the cauda differ in post-thaw motility or membrane integrity [29]. Canine epididymal spermatozoa were more resistant to vitrification than ejaculated spermatozoa [30]. In contrast to the cat, in the dog it seems, however, that sperm quality after cryopreservation often is inferior to that of ejaculated spermatozoa [15, 31–33]. However, direct comparisons within the same studies are lacking.

### Changes at ejaculation and the effect of exposure to seminal plasma

The cauda epididymal environment maintains spermatozoa in a quiescent state, supporting long-term viability. Low Ca^2+^ concentrations and acidic pH prevent premature capacitation and acrosome reaction [34]. The environment of the spermatozoa rapidly changes at ejaculation, with exposure to fluids from the accessory sex glands. This result in dilution of the spermatozoa, increase in pH, increase in Na^+^ and Cl^−^ concentrations, and a rapid change in the osmotic environment. Exposure to seminal plasma (SP) plays a central role in modulating sperm function [35]. Seminal plasma is a mixture of secretions from the epididymis and the accessory glands. In dogs, the prostate is the only major accessory gland, making prostatic fluid (PF) the principal SP component [36]. In cats, bulbourethral secretions dominate, with PF comprising most of the remainder [37]. Unlike in dogs, PF is alkaline in cats, while feline SP overall is not [37]. SP contains decapacitation factors that may stabilize sperm membranes by masking surface antigens and zona-binding receptors [38]. Spermatozoa from the canine epididymal cauda possess for example a progesterone receptor that is of importance for the acrosome reaction. This receptor is masked in ejaculated spermatozoa exposed to seminal fluids, and revealed again after capacitation [39]. This suggests that ejaculated spermatozoa may achieve greater stability and longevity, being protected from premature capacitation-related membrane changes. Conversely, SP can also destabilize sperm membranes, as it contains both capacitation-inhibiting [40–42], and capacitation-enhancing factors [43, 44]. In dogs, addition of PF to epididymal spermatozoa prior to freezing has yielded beneficial effects on sperm motility and viability [15, 33]. Other studies reported that the addition of PF to canine spermatozoa activates sperm motility in fresh spermatozoa, but had negative effects on post-thaw semen quality [31]. In cats, post-thaw exposure of epididymal spermatozoa SP reduced semen quality [45]. Presence of seminal plasma may, however, be important for fertilization after insemination. Conception rate and number of newborn after artificial insemination were significantly higher when canine caudal epididymal spermatozoa had been exposed to PF, compared with unexposed epididymal spermatozoa [15, 46]. The increased fertility when seminal plasma is added may not only be related to sperm function, but could also be related to modulation of uterine contractions [47].

### Freezability and fertility of spermatozoa collected from different epididymal regions

Although both pre-freeze and post-thaw motility are lower, spermatozoa from the feline corpus region exhibit similar freezability to those from the cauda [27, 48]. Spermatozoa from all macroscopic regions of the feline epididymis (caput, corpus, and cauda) are capable of undergoing capacitation and acrosome reaction in vitro in vitro. However, caudal spermatozoa required a shorter incubation time compared with those from the caput and corpus, indicating that they capacitate more readily [18]. Frozen–thawed spermatozoa from the corpus and cauda displayed comparable fertilizing ability in vitro, although blastocyst development rates were lower when corpus spermatozoa were used [49]. Including corpus spermatozoa in feline sperm recovery would increase the total number of retrievable spermatozoa, and thereby the number of AI doses. By contrast, spermatozoa from the caput are unsuitable for AI or IVF due to their low motility. In the dog, the corpus epididymidis is characterized by low progressive motility and histological features of the middle segment, a region primarily associated with sperm maturation rather than storage [20]. Consequently, canine corpus spermatozoa are likely less suitable for cryopreservation for AI or IVF, compared with corpus spermatozoa from the feline epididymis. In conservation biology, intracytoplasmic sperm injection (ICSI) may be a viable alternative, as it does not require motile spermatozoa. However, neither ICSI nor IVF are currently applied to domestic dogs or cats in clinical practice.

### Collection methods

The method of epididymal sperm retrieval can influence post-thaw semen quality. The simplest approach is mincing the organ in collection medium, allowing spermatozoa to migrate from the tissue before removal of debris [16, 31]. However, contamination with blood and tissue is inevitable with this technique. Retrograde flushing of the vas deferens yields higher-quality spermatozoa in the dog compared with mincing, although this technique may be challenging in cats due to the small duct lumen, and even in medium-sized dogs such as Beagles [50–52]. Percutaneous epididymal sperm aspiration (PESA) has been evaluated in dogs [53–55]. In a small cohort (*n* = 3), PESA induced transient antisperm antibodies but no long-term adverse effects on sperm output or motility [54]. In rats, however, repeated PESA has been associated with inflammation, fibrosis, and epididymal granulomas [56, 57]. Moreover, PESA yields lower sperm counts in dogs than retrograde flushing or mincing [53, 55]. Given that the epididymis is a single convoluted duct, PESA should be considered a risky approach for fertility preservation in valuable breeding dogs. In human medicine, PESA is used to obtain epididymal spermatozoa for ICSI in men with obstructive azoospermia [58], a condition that is relatively uncommon in dogs, where non-obstructive azoospermia predominates [59]. Other methods include aspiration of epididymal fluid post-castration, which appears ineffective in cats (author’s unpublished observations) and inferior to mincing in dogs [60]. Sperm retrieval by squeezing the cauda into a Petri dish using an anatomic clamp has also been described [61].

### The effect of time and temperature

Following castration or death, epididymal sperm quality declines over time. If immediate collection and freezing are not possible, refrigeration of the epididymis preserves sperm quality more effectively than storage at ambient temperature [62, 63]. Canine epididymides stored at room temperature for up to 6 h showed no significant decline in quality [63, 64]. However, after 12 h, storage at 4 °C preserved sperm function better than at room temperature [63]. In cats, epididymides stored at 5 °C for 2 days yielded spermatozoa of comparable quality to those extended in a Tris–egg yolk medium [65]. After 4 days, however, epididymal storage was associated with increased tail abnormalities compared with diluted samples stored under the same conditions [65]. Similarly, keeping cat epididymal spermatozoa at 5 °C in a Tris-glucose-20% egg yolk extender for 24 h prior to freezing, did not impair the freeze-thaw process [66].

### Fertility after AI with frozen-thawed epididymal spermatozoa

Reports of fertility outcomes after AI with frozen–thawed epididymal spermatozoa in dogs and cats remain limited. The first live births of kittens from AI with frozen–thawed epididymal spermatozoa were reported in 2003 [67]. Despite comparable sperm motility and viability to ejaculated spermatozoa, conception rates were lower [67, 68]. AI is performed far less frequently in cats than in dogs, and there are no clinical reports of AI with feline epididymal spermatozoa.

In the dog, most published data derive from experimental surgical AI using frozen–thawed epididymal spermatozoa, which achieved a pregnancy rate of 21.7% (13/60 bitches) [15, 52, 69]. Case reports also exist, including the first successful AI resulting in a single Boxer puppy [70], and a more recent report of eight puppies born following transcervical AI with frozen–thawed epididymal semen [1]. In both cases, semen collection by ejaculation was not feasible due to acute deterioration in health.

#### Concluding remarks and summary

Epididymal spermatozoa should be retrieved as soon as possible after orchidectomy or the death of the male. If immediate sperm collection and cryopreservation cannot be performed, the organs should be refrigerated, but not frozen, to preserve sperm viability. To prevent dehydration, they should be placed in a non-toxic, airtight plastic bag. Alternatively, spermatozoa can be released into an appropriate extender and stored refrigerated until cryopreservation is feasible.

Sperm retrieval by epididymal mincing is the simplest and most rapid method but is associated with contamination by blood and tissue debris; therefore, visible blood vessels should be carefully removed before processing. Retrograde flushing of the epididymal duct can reduce tissue contamination, and may particularly be useful in larger dogs.

For artificial insemination, spermatozoa from the cauda epididymidis are most suitable in the dog, whereas both corpus and cauda spermatozoa can be used in the cat. In conservation contexts, where intracytoplasmic sperm injection (ICSI) may be applied, spermatozoa from all epididymal segments, and even testicular spermatozoa, could be of potential value.

The possible advantages of supplementing epididymal sperm with seminal plasma remain unclear. However, in most cases requiring epididymal sperm collection, simultaneous retrieval of seminal fluid from the same male is not feasible. Extenders and cryopreservation protocols used for epididymal spermatozoa are generally those established for ejaculated samples.

## Data Availability

Not applicable.

## Abbreviations

AI: Artificial insemination
ICSI: Intracytoplasmic sperm injection
IVF: In vitro fertilization
PESA: Percutaneous epididymal sperm aspiration
PF: Prostatic fluid
SP: Seminal plasma

## Declarations

### Authors’ information

Eva Axnér graduated in Veterinary medicine in 1991 from the Swedish University of Agricultural Sciences (SLU). She achieved her PhD-degree in Obstetrics and Gynaecology at Faculty of Veterinary Medicine, SLU 2000, on the subject of feline epididymal sperm morphology and sperm maturation. In 2002, Eva Axnér became Diplomate of the European College of Animal Reproduction (Dipl. ECAR, small animals), and Professor in carnivore reproduction 2012. At present, she is working as a university teacher and researcher in addition to clinical practice at the university animal hospital. She is responsible for the small animal fertility clinic at SLU. Her research interests lie in carnivore reproduction with a special emphasis on semen conservation, spermatology, artificial insemination, and feline welfare in relation to reproduction.
